# Priorities and Perspectives Regarding Goals and Outcomes of Support for Autistic Children Under 12 Years: A Systematic Review

**DOI:** 10.1177/13623613261433132

**Published:** 2026-04-20

**Authors:** Phoebe Jordan, Hannah Waddington, Matt Hammond, Mirko Uljarevic, Willow J Sainsbury, Jessica Tupou

**Affiliations:** 1 Victoria University of Wellington, New Zealand; 2 Stanford University, USA; 3 University of Auckland, New Zealand

**Keywords:** autism, goal setting, neurodiversity-affirming, stakeholder perspectives, support priorities

## Abstract

Autistic individuals, family members, and professionals often hold differing perspectives on the goals and outcomes of supports for autistic children under 12 years. While traditional approaches prioritise the acquisition of neurotypical behaviours, emerging frameworks emphasise autonomy, self-determination, and well-being. This systematic review synthesised findings from 15 studies using qualitative, quantitative, and mixed-methods designs, which were assessed for methodological quality using the Joanna Briggs Institute checklists. Communication, social inclusion, and child well-being emerged as shared priorities. Notable differences were observed; professionals tended to focus on normative developmental goals such as skill acquisition and behavioural compliance, while autistic individuals and family members more often valued flexibility, self-advocacy, and strengths-based approaches. Tensions persist between medicalised and neurodiversity-affirming paradigms. To ensure supports align with what matters to autistic people, future research should prioritise co-design with autistic individuals and families, embrace cultural responsiveness, and develop tools that can flexibly but consistently assess neurodiversity-affirming outcomes. These steps will support more ethical, inclusive, and meaningful goal-setting practices in autism research and support.

**Lay Abstract:**

Autistic children, their families, and the people who support them often want different things from autism services. Some approaches still focus on teaching autistic children to behave more like non-autistic children, such as making eye contact or using spoken language. However, many autistic people and families are calling for support that values autistic ways of being and prioritises well-being, comfort, and meaningful participation. This systematic review brought together findings from 15 research studies published in the last 10 years. These studies explored what goals matter most to autistic adults, parents, and professionals when supporting autistic children aged 0–12. We reviewed studies that used interviews, surveys, or mixed methods and assessed their quality using standard research checklists. Across studies, several shared priorities emerged. Communication was important to everyone, but in broad terms supporting children to express themselves in the ways that work best for them, including through alternative augmentative communication or non-spoken communication. Stakeholders also consistently valued children’s emotional well-being, mental health, and feeling safe and understood. Many studies highlighted the importance of autonomy, including supporting children to make choices, develop a sense of identity, and have control in their daily lives. Traditional goals such as reducing autistic traits, encouraging eye contact, or teaching neurotypical social skills were often rated as less important. There was strong agreement that supports should help children build comfort, confidence, and inclusion rather than force conformity.

Autism is a natural variation of human neurology, characterised by diverse ways of thinking, communicating, and experiencing the world (Bottini et al., 2024). Historically, however, it has often been framed through a medical lens as a disorder requiring intervention to reduce perceived deficits and promote neurotypical development (Chellappa, 2023). This framing has shaped the support goals prioritised by different groups. Traditional approaches have often emphasised behavioural modification, focusing on goals such as increased eye contact, reduced stimming, and alignment with peer norms (Kapp, 2019; Turnock et al., 2022). In contrast, the neurodiversity paradigm views autism as an inherent aspect of human diversity, shifting the focus towards autonomy, adaptive skills, well-being, and supportive environments (Bottini et al., 2024; Kapp, 2019). This framing aligns with the perspectives of many autistic individuals and neurodiversity advocates who have participated in research, most of whom favour supports that enhance quality of life and accessibility rather than enforcing conformity (Hersh et al., 2024). However, autistic perspectives are not monolithic, and research to date may underrepresent individuals who are unable to participate due to study design or accessibility barriers. This systematic review examines the goals and outcomes prioritised for autistic children by autistic individuals, family members, and professionals. It compares traditional approaches, often focused on behavioural compliance and skill acquisition, with neurodiversity-affirming approaches that emphasise autonomy, well-being, and inclusive participation. The review identifies areas of alignment, tension, and opportunities to centre stakeholder values in more inclusive goal-setting practices.

## Conceptual Approaches and Stakeholder Perspectives

Although some authors distinguish between “goals” and “outcomes,” the terms are often used interchangeably. This review uses the combined term “goals/outcomes” to refer to both immediate support aims and longer-term developmental aspirations (Sulek et al., 2024).

Conceptualisations of autism shape stakeholder priorities for support and related goals/outcomes. Traditionally, interventions like applied behaviour analysis and early intensive behavioural support have emphasised skill acquisition and the reduction of socially atypical behaviours (Nahmias et al., 2019). Guided by a medical model, these approaches focus on reducing autistic traits, promoting independence, and improving communication goals still valued by many professionals and some parents (Monz et al., 2019). Proponents of this model often view independence and communication gains as indicators of successful adaptation and participation in society. In contrast, neurodiversity-affirming approaches conceptualise participation and communication differently: rather than measuring success by proximity to neurotypical norms, they prioritise comfort, authenticity, and autonomy within diverse ways of being. From this perspective, independence is not defined by doing things without support, but by having choice and agency within one’s environment; likewise, communication is valued in whatever form best enables self-expression and connection. These differing assumptions reflect broader philosophical divides between deficit-based models seeking to remediate difference and affirming frameworks seeking to accommodate it. Some members of the autistic community have critiqued behaviour-based goals/outcomes for prioritising typicality over personal flourishing (Hersh et al., 2024).

Naturalistic developmental behavioural interventions (NDBIs), such as the Early Start Denver Model and Pivotal Response Treatment, emerged as a response to critiques of classic early intensive behavioural interventions, integrating structured behavioural strategies with developmental and play-based methods. These approaches prioritise child-led engagement, shared affect, and naturalistic reinforcement and have been associated with improvements in communication, adaptive functioning, and social reciprocity across meta-analyses (Nahmias et al., 2019). Many parents value NDBIs for their emphasis on joint attention and early social interaction, and for appearing less rigid than earlier behaviourist models (Mirenda et al., 2022). However, some autistic individuals have raised concerns that “success” in these models is often benchmarked against neurotypical developmental milestones, such as speech, eye contact, or specific play behaviours, rather than supporting alternative pathways to autonomy and connection (Schuck et al., 2022). For instance, while goals/outcomes like tying shoelaces or increasing daily living skills are often framed as essential for independence, the underlying assumptions about what “independence” should look like can overlook diverse ways of being or undervalue interdependence. These tensions are further complicated for autistic children with intellectual disabilities, who are frequently underrepresented in research and may require higher-support needs (Pellicano & Den Houting, 2021). These children may benefit from structured support approaches but still require careful consideration of whether goals/outcomes align with the child’s preferences, capacities, and well-being.

Tensions remain in how goals/outcomes are determined. Clinicians and caregivers have traditionally led goal/outcome setting, often prioritising skills aligned with neurotypical developmental milestones, such as speech and specific social behaviours (Waddington et al., 2023). In contrast, the autistic community have increasingly advocated for goals/outcomes centred on autonomy, self-defined progress, and well-being. While these principles are often associated with verbal self-advocacy, they are equally important for autistic individuals with limited spoken language or intellectual disabilities. For these individuals, autonomy may be expressed through choices, preferences, behaviours, or supported decision-making, and meaningful progress can be defined through increased comfort, communication access, and quality of life (Jaswal & Akhtar, 2018). Recognising these diverse ways of expressing agency is essential for setting goals/outcomes that are truly neurodiversity-affirming and inclusive of all support needs.

Communication and social interaction goals/outcomes illustrate these differences. Supports have traditionally prioritised spoken language and discouraged scripting, echolalia, or alternative augmentative communication (AAC) (Blanc et al., 2023; Donaldson et al., 2021). While some parents still view spoken language as key to independence, many autistic advocates value diverse forms of communication, including AAC, scripting, and text-based methods (Adams & Gaile, 2020). Likewise, social goals have often emphasised eye contact, small talk, and group participation, yet some autistic people express preferences for deep conversation, parallel play, or online interactions (Morris et al., 2024; Zolyomi et al., 2017).

Autistic voices have historically been marginalised in support planning, resulting in goals/outcomes that aim to reduce autistic traits rather than enhance quality of life (Graf-Kurtulus & Gelo, 2024). Outcome measures have often overlooked priorities such as sensory comfort, autonomy, and inclusion (Roche et al., 2020; Schiltz et al., 2024). However, autistic-led advocacy and participatory research are now reshaping the landscape. When autistic people shape research agendas, goals/outcomes prioritise mental health, autonomy, and inclusive environments, moving away from externally imposed behavioural norms (Roche et al., 2020). The “Nothing about us, without us” movement calls for co-production in research and practice, reinforcing a shift towards support systems that affirm autistic agency, participation, and thriving (Pellicano & Den Houting, 2021).

Although there is no universally accepted definition, neurodiversity-affirming principles generally emphasise respecting neurological diversity as a natural form of human variation, prioritising environments that adapt to the individual rather than requiring individuals to conform (Chapman & Botha, 2022; Leadbitter et al., 2021). Across frameworks, these principles commonly include recognising autism as an identity rather than a disorder, supporting autonomy and self-determination, valuing diverse communication styles, promoting sensory and emotional well-being, and fostering inclusion and belonging. The present review draws on these conceptual threads to examine how different stakeholders, autistic individuals, family members, and professionals define meaningful goals/outcomes for autistic children.

Against this backdrop, the present review explores how autistic individuals, family members, and professionals prioritise support goals/outcomes for autistic children aged 0–12 years. This review seeks to explore these perspectives by addressing the following questions: (a) what goals/outcomes do family members, professionals, and autistic adults identify as priorities for autistic children? (b) to what extent do neurodiversity-affirming principles feature in the goals/outcomes identified by these groups? and (c) how do the perspectives of family members, professionals, and autistic adults differ or align regarding goals/outcomes for autistic children?

## Methodology

### Design

This systematic review followed the Preferred Reporting Items for Systematic Reviews and Meta-Analyses 2020 guidelines to ensure clarity and rigour (Page et al., 2021; see Supplementary Materials 1 for the PRISMA checklist). The review protocol was preregistered with the Open Science Framework and is publicly available at https://osf.io/rqhdb/overview. No changes were made to the protocol after registration. The review focused on empirical studies and used a neurodiversity-affirming framework to assess how the goals/outcomes identified by family members, professionals, and autistic adults reflected affirming practices.

### Search Strategy

A comprehensive electronic search was conducted between January and March 2025 across four databases: CINAHL, PubMed, Scopus, and Google Scholar (limited to the first 100 results). The following Boolean search terms were used to maximise relevant results: (“autis*” OR “ASD”) AND (“child*” OR “youth*”) AND (“goal*” OR “outcome*” OR “target*”) AND (“perspectiv*” OR “priorit*” OR “view*” OR “opinion*”).

All search results were imported into Covidence, a web-based tool for managing systematic reviews. Covidence was used to remove duplicates and manage the screening and data extraction process. Additional studies were identified through ancestral searching (reviewing reference lists of relevant papers and systematic reviews) and citation searching. One article (Schuck et al., 2024) was identified during citation searching after initial data extraction, as it was not found in the original database results.

### Eligibility Criteria

Studies were included in the review if they met the following criteria:

1. Included family members, professionals, and/or autistic adults reflecting on goals and outcomes for autistic children aged 0–12 years. The age cap of 12 years was selected because research shows significant shifts in goals/outcomes during adolescence, reflecting changes in developmental priorities, education, and social integration (Sosnowy et al., 2017). Autism supports in early and middle childhood typically emphasise foundational skills, such as communication, social engagement, and adaptive living skills. In adolescence, priorities shift towards vocational skills, daily living independence, social interactions, and self-determination (Will et al., 2018), warranting separate analyses for older populations.
2. Examined priorities and perspectives regarding goals/outcomes identified as priorities or relevant for autistic children.
3. Employed empirical qualitative, quantitative, or mixed-methods research.
4. Were published within the last 10 years (2015–2025), to ensure relevant and up-to-date information.
5. Were written in English in a peer-reviewed journal.

### Study Selection

The study selection process was conducted in two phases using Covidence. First, title and abstract screening was performed by the first author (P.J.), where articles were independently reviewed based on their relevance to the inclusion criteria. The fifth author (W.J.S.) completed interrater agreement on 20% of the title and abstract search, articles chosen by random (94%) with a kappa statistic of 0.73. Next, full-text screening was conducted by W.J.S. for potentially relevant studies, with final inclusion decisions recorded. The fifth author (W.J.S.) completed interrater agreement on 20% of the full-text review, articles chosen by random (90%) with a kappa statistic of 0.73. Any discrepancies were resolved through discussion between P.J. and W.J.S. until consensus was reached.

### Data Extraction

A structured data extraction table was used to systematically summarise key information from each study, categorising data by study, methodology, data collection methods, and analytical strategies. The table also captured prioritised goals/outcomes for autistic children, highlighting any variations across stakeholder groups. W.J.S. completed interrater agreement on 20% of the articles, articles chosen by random for data extraction (89%).

We developed a structured framework to evaluate how each study’s conceptualisation, design, and procedures aligned with neurodiversity-affirming principles (see Supplementary Materials 2). We grounded this framework in an extensive literature review, identifying five core neurodiversity-affirming principles: (a) adopting a strengths-based focus (Leinfuss & O’Hara, 2024); (b) supporting self-determination and autonomy (Ryan et al., 2024); (c) adapting environments and upskilling allistic individuals (Waddington et al., 2023); (d) valuing diverse communication methods (Gaddy & Crow, 2023); and (e) respecting sensory and processing differences (Morgan, 2019). Each study received a rating indicating whether it positively endorsed these principles (Yes), negatively represented them (No), or did not discuss them (N/A, applicable only to Principles 4 and 5). Ratings were based specifically on researcher choices about study design, stated goals, procedures, and outcomes, rather than on participants’ expressed views or observed behaviours.

### Data Synthesis

Descriptive analysis was used to summarise study characteristics and findings related to goal/outcome priorities, which are presented in tables. A narrative synthesis was then conducted to identify key patterns and trends across stakeholder perspectives. Studies were grouped by the perspectives they explored (e.g. parents, autistic adults, educators, professionals) and how each group prioritised different goals/outcomes. The frequency of key themes was quantified (e.g. “10 out of 15 studies prioritised alternative communication supports”). Comparisons were also made between stakeholder groups to highlight areas of agreement and difference in their priorities.

### Quality Assessment

Study quality was assessed using the Joanna Briggs Institute (JBI) Critical Appraisal Checklists (Munn et al., 2020). The JBI checklists were selected for their flexibility across qualitative, quantitative, and mixed-methods designs, and for their focus on assessing methodological rigour without privileging any specific research paradigm. Qualitative studies were evaluated using the Qualitative Research Checklist, and quantitative studies using the Analytical Cross-Sectional Checklist. Mixed-methods studies were assessed according to their primary data type, determined based on the dominant form of data collection and reporting in the results, for example, studies that primarily presented and analysed quantitative survey data were appraised using the quantitative checklist. W.J.S. completed interrater agreement on 20% of the articles, articles chosen by random for quality assessment (100%) and kappa was 1.0.

### Community Involvement

This review was led by P.J., an autistic adult with professional involvement in supporting autistic individuals and their families. P.J. conceptualised and conducted the review, including developing the neurodiversity-affirming framework used to evaluate studies. H.W. and J.T. brought both lived experience as family members of autistic individuals and extensive professional expertise in clinical and community-based autism supports. W.J.S., an academic in the autism field, contributed feedback on the framework and interpretation of findings. M.U. offered expertise in developmental psychopathology, person–environment fit, and autism heterogeneity, while M.H. contributed a background in social psychology, particularly regarding identity, stereotype threat, and inclusive measurement. The development and interpretation of this review reflect a collaborative process grounded in lived experience, neurodiversity-informed scholarship, and clinical-practical knowledge, with the goal of promoting more inclusive, affirming approaches to autism research and support.

## Results

### Study Selection

**Figure 1.**
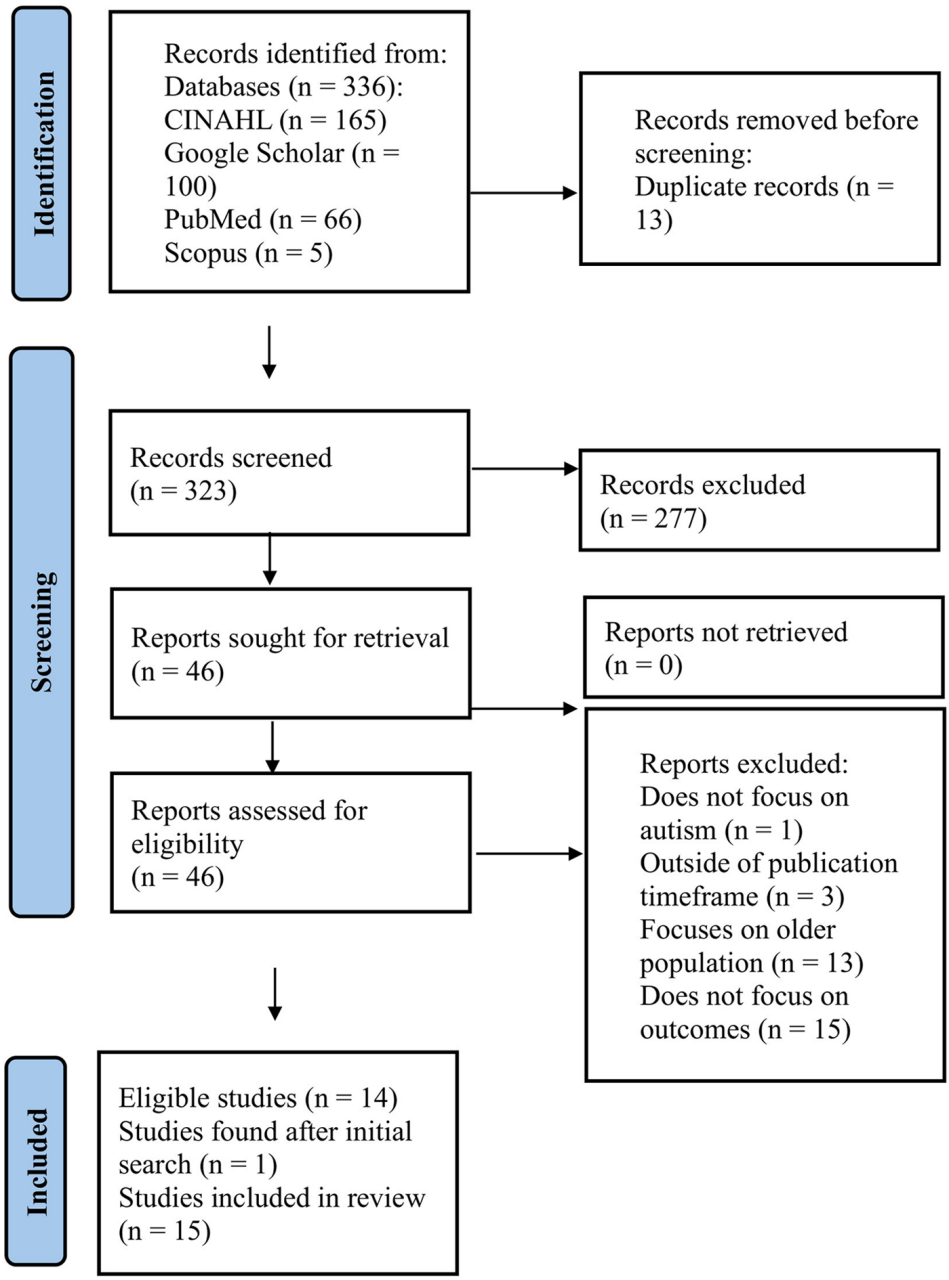
PRISMA flow diagram.

A total of 336 records were identified through database searches (CINAHL = 165, Google Scholar = 100, PubMed = 66, Scopus = 5). After removing 13 duplicates via Covidence, 323 records remained for title and abstract screening. Of these, 277 were excluded based on relevance. Forty-six full-text articles were assessed for eligibility, and 32 were excluded for the following reasons: did not focus on outcomes (*n* = 15), wrong participant population (*n* = 13), outside of publication timeframe (*n* = 3), and did not focus on autism (*n* = 1) (see Supplementary Materials 3). Fourteen studies met the inclusion criteria through initial database and ancestral searches. One additional study (Schuck et al., 2024) was identified through citation searching after the initial extraction phase, bringing the final number of included studies to 15. The PRISMA flow diagram is presented in Figure 1.

### Study Characteristics

This literature review synthesises findings from 15 studies exploring stakeholder perspectives on the goals/outcomes for autistic children. Table 1 summarises key study and participant characteristics, including study design, sample size, data collection and analysis methods, participant roles, and focus population. Supplementary Materials 4 provides additional demographic and contextual information, such as geographical location, participant gender and ethnicity, and further role-specific details.

**Table 1.** Study and participant characteristics.

Author(s) and year	Study design and data collection	*n*	Analysis	Participant type			Focus population
				Autistic adults	Parents	Prof.	
Bent et al. (2024)	Mixed-methods survey	238	Likert-scale responses and thematic analysis	Yes	Yes	Yes	Infants (0–2 years) likely to be autistic.
Brock et al. (2019)	Quantitative survey	99	Statistical analysis	No	No	Yes	Autistic students age NS
Clark & Adams (2020)	Mixed-methods questionnaire	134	Q-sort ranking and discussion	No	Yes	No	Autistic students aged 6–17
Derguy et al. (2015)	Qualitative interviews	162	Qualitative content analysis	No	Yes	No	Autistic children aged 3–10
De Korte et al. (2022)	Qualitative interviews	149	Thematic analysis	No	Yes	No	Autistic children aged 2–6
DuBay et al. (2018)	Mixed-methods survey	55	Quantitative and thematic qualitative analysis	No	Yes	No	Young autistic children
Gormley et al. (2024)	Mixed-methods survey	109	Thematic analysis	Yes	No	Yes	Autistic students age NS
Laubscher et al., 2024	Qualitative interviews	8	Thematic analysis	No	Yes	No	Young autistic children
Lindsay et al. (2016)	Mixed-methods interviews	129	Likert-scale ratings and qualitative elaborations	No	Yes	No	Autistic children aged 5–12
Petrina et al. (2015)	Quantitative survey	74	Data analysed using Mann–Whitney U tests and Friedman two-way ANOVA	No	Yes	No	Autistic children aged 5–10
Pfeiffer et al., 2016	Qualitative interviews	10	Thematic analysis	Yes	Yes	Yes	Autistic children age NS
Schuck et al. (2024)	Qualitative survey	214	Thematic analysis	Yes	No	No	Autistic children age NS
Sulek et al. (2024)	Quantitative survey	181	Statistical and thematic analysis	Yes	Yes	Yes	Autistic children age 0–5 years and 6–12 years separately
Waddington et al. (2023)	Qualitative survey	122	Thematic analysis	Yes	Yes	Yes	Autistic children under 6
Waddington et al. (2024)	Quantitative survey	326	Statistical and thematic analysis	Yes	Yes	Yes	Autistic children under 6

Prof: professional; NS: not specified.

The 15 included studies used 7 qualitative, 4 quantitative, and 4 mixed-methods designs. Data collection methods comprised eight surveys and seven interviews. Research was conducted across eight countries: Australia and the United States (five studies each), New Zealand (four), the United Kingdom and Canada (two each), and the Netherlands, France, and Ireland (one each), with some studies spanning multiple locations. Participants included autistic adults (eight studies), parents or caregivers (12), and professionals (seven, including educators, clinicians, and researchers). Four studies involved only one stakeholder group, while 11 included two or more. Most studies reported demographic data, with 13 reporting gender; most samples were predominantly women, particularly among parent participants, with some more recent studies including non-binary participants. Nine studies reported ethnicity, with most participants identifying as White or European, though some studies included representation from Māori, Pacific, Asian, and mixed-race communities. Focus populations included autistic or high likelihood of being autistic infants (one study), young children under six (five), school-aged children (four), and young students of unspecified ages (four). In the latter group, the term “young” was used by the authors and was interpreted as referring to children under 12 years of age, consistent with the review’s inclusion criteria. The majority of studies (60%) were recruited online through social media and online flyers.

### Quality Assessment

Quantitative studies scored between 6/8 and 8/8, with Sulek et al. (2024) and Waddington et al. (2024) receiving full marks. Qualitative studies scored between 7/10 and 10/10, with De Korte et al. (2022), Pfeiffer et al. (2016), Waddington et al. (2023), and Schuck et al. (2024) scoring highest (10/10). Most studies showed strong alignment between aims and analytical methods, though five lacked details on researcher positionality or influence (see Supplementary Materials 5 and 6).

### Identified Goals and Priorities

Table 2 summarises the number of studies covering higher and lower priority goals/outcomes. See Supplementary Materials 9 for detailed table on studies’ participants and higher and lower priorities on goals/outcomes.

**Table 2.** Number of studies prioritising higher and lower goals/outcomes.

	Number of studies which identified this goal/outcome as higher priority = *n* (%)
Communication	7 (46.7)
Child mental health and well-being	7 (46.7)
Autonomy and self-determination	6 (40)
Support for parents and professionals	3 (20)
Community inclusion and social acceptance	3 (20)
	Number of studies which identified this goal/outcome as lower priority = *n* (%)
Reducing autistic traits	4 (26.7)
Play and neurotypical social skills	3 (20)
Academic skills	1 (6.7)

#### Higher-Priority Goals/Outcomes

Across the reviewed studies, several goals/outcomes were consistently prioritised, particularly in the areas of communication, mental well-being, autonomy, and inclusive participation. Communication was prioritised in seven studies, with a general emphasis on diverse and functional strategies rather than focusing solely on spoken language. While DuBay et al. (2018) explicitly prioritised spoken language, other studies, such as Brock et al. (2019), Gormley et al. (2024), Laubscher et al. (2024), and Waddington et al. (2023), highlighted broader communication goals. De Korte et al. (2022) valued improvements in social communication, and Waddington et al. (2024) identified communication as a moderate, but not top, priority. Schuck et al. (2024) further reinforced the importance of recognising diverse autistic communication methods and cautioned against privileging speech over alternatives like AAC or scripting.

Child mental health and well-being were high-priority goals/outcomes in eight studies. Sulek et al. (2024) identified mental well-being as the top-rated goals/outcomes across stakeholder groups, while Gormley et al. (2024) reported strong endorsement for physical and mental health, and emotional awareness. Bent et al. (2024) prioritised infant quality of life and emotional support. In Clark and Adams (2020), both Q-sort data and survey responses ranked child anxiety, health, and well-being as central concerns. Similarly, De Korte et al. (2022) and Laubscher et al. (2024) emphasised emotional well-being, and earlier work by Lindsay et al. (2016) and Petrina et al. (2015) highlighted emotional development as key goals/outcomes.

Autonomy and self-determination emerged as key goals/outcomes across six studies. Gormley et al. (2024) reported strong support for self-determination, while Bent et al. (2024) emphasised enabling infant autonomy and adapting environments to support it. Waddington et al. (2023, 2024) identified both self-determination and authentic autistic identity as top priorities. Pfeiffer et al. (2016) highlighted long-term independence and inclusive participation, and Schuck et al. (2024) underscored the importance of autonomy, self-advocacy, and interdependence, rejecting forced independence and framing autonomy as central to autistic well-being.

Support for parents and professionals was identified as a key goal/outcome across three studies, aimed at improving outcomes for autistic children. Bent et al. (2024) highlighted strong endorsement for parent education on autism and neurodivergence, noting that informed, confident parenting supports stronger developmental outcomes. Derguy et al. (2015) found that parents prioritised consistent support from trained professionals and institutions to ensure stable, high-quality care. Clark and Adams (2020) similarly emphasised the importance of staff education and support in schools to create environments where autistic children can thrive.

Community inclusion and social acceptance emerged as key priorities across three studies. Clark and Adams (2020) found that parents valued greater community awareness and understanding of autism. Pfeiffer et al. (2016) reported that autistic adults prioritised social acceptance and inclusive participation, underscoring the need for goals/outcomes that promote meaningful societal inclusion for autistic children. Schuck et al. (2024) reinforced this perspective, rejecting normalisation-based goals and advocating for recognition and appreciation of autistic ways of being.

#### Lower-Priority Goals/Outcomes

Across the reviewed studies, several goals/outcomes were less likely to be rated as high priorities by participants, particularly those related to reducing autism characteristics, neurotypical social skills, and academic achievement. While not always explicitly rejected, these goals/outcomes tended to receive lower endorsement or were ranked among the least important.

Goals/outcomes aimed at reducing autistic traits were among the most consistently rated as lower in priority across four studies. For example, both Waddington et al. (2023) and Sulek et al. (2024) found that autistic adults, parents, and professionals frequently rated these types of goals/outcomes as among the least important and most likely to be inappropriate. Waddington et al. (2024) ranked reducing autism characteristics as the lowest-priority goal/outcome and Bent et al. (2024) found low endorsement for reducing repetitive behaviours and encouraging eye contact. Schuck et al. (2024) explicitly identified reducing stimming (motor and vocal) and emphasising normalisation or neurotypical standards as widely unacceptable goals/outcomes.

Goals/outcomes related to play and neurotypical social skills were also less frequently prioritised. Pretend play, structured social skills training, and efforts to make play more neurotypical were rated among the lowest-priority outcomes and most likely to be inappropriate in Waddington et al. (2023) and Sulek et al. (2024), while participants in Waddington et al. (2024) ranked play skills near the bottom. Schuck et al. (2024) further reinforced that goals/outcomes oriented towards neurotypical social expectations were consistently criticised as undermining autistic identity. Only Waddington et al. (2024) ranked academic skills among the lowest-priority goals/outcomes.

### Similarities and Differences in Perspectives across Stakeholders

Supplementary Materials 7 summarises the similarities and differences in perspectives across participant groups in the 10 studies that included multiple stakeholder or comparison groups. Across these studies, there was broad alignment across stakeholder groups on the importance of well-being, autonomy, and inclusive practices, especially among autistic adults and professionals. Autistic participants consistently prioritised self-determination, authentic communication, and environmental adaptation, while rejecting deficit-based or behaviour-focused goals/outcomes (Sulek et al., 2024; Waddington et al., 2023). Participatory studies highlighted strong support from autistic adults for respecting identity and boundaries, including rejecting enforced eye contact and neurotypical social skills (Gormley et al., 2024; Waddington et al., 2023).

Parent perspectives were more variable. Some parents emphasised long-term planning, emotional development, or educational progress (Brock et al., 2019; Pfeiffer et al., 2016). In DuBay et al. (2018), caregivers prioritised spoken communication and structured support, while also noting a lack of culturally appropriate and bilingual resources.

In more recent studies, professionals increasingly aligned with neurodiversity-affirming values. Educators in Gormley et al. (2024) valued communication, daily living skills, and social inclusion over academic targets, reflecting a shift towards holistic, functional outcomes. Sulek et al. (2024) similarly found that professionals rejected behaviour-reduction goals, instead emphasising mental health, accessibility, and inclusion. Bent et al. (2024) reported strong support for parent education and environmental adaptation, though some participants still endorsed early diagnostic labelling, highlighting ongoing tensions in defining appropriate outcomes. In contrast, older studies such as Brock et al. (2019), Derguy et al. (2015), and Petrina et al. (2015) prioritised academic, behavioural, or standardised developmental goals, with limited attention to well-being, autonomy, or environmental change.

### Alignment with Neurodiversity-Affirming Principles

Supplementary Materials 8 summarises alignment with five core neurodiversity-affirming principles: (a) strengths-based focus, (b) self-determination and autonomy, (c) adapting environments and upskilling others, (d) diverse communication, and (e) sensory and processing differences.

Eleven studies included a strengths-based focus, emphasising children’s interests or developmental outcomes considered positive within each study, such as enhanced well-being, emotional growth, or greater participation in meaningful activities. Seven explicitly supported self-determination, highlighting choice-making, authentic identity, and interdependence. Nine studies prioritised adapting environments, through caregiver education, inclusive settings, or structural supports, though some retained traditional behaviour-focused elements. Five studies valued diverse communication methods such as AAC, especially in participatory designs. Seven studies recognised sensory and processing differences, promoting goals/outcomes to improve sensory accessibility and support regulation.

### Temporal Trends in Neurodiversity-Affirming Perspectives

Across the literature, pre-2020 studies tended to prioritise goals/outcomes framed around skills training, behaviour management, or normalising development such as improving social skills, managing challenging behaviour, and advancing academic or developmental milestones (Brock et al., 2019; Derguy et al., 2015; DuBay et al., 2018; Lindsay et al., 2016; Petrina et al., 2015). In contrast, post-2020 studies increasingly centre autonomy, well-being, environmental adaptation, and neurodiversity-affirming priorities, highlighting the importance of inclusion, communication access, self-determination, and supporting children in ways that respect autistic identity (Bent et al., 2024; Clark & Adams, 2020; De Korte et al., 2022; Gormley et al., 2024; Laubscher et al., 2024; Schuck et al., 2024; Waddington et al., 2023, 2024). Supplementary Materials 9 provides detailed summaries of each study.

## Discussion

This review analysed 15 studies examining how stakeholders prioritise support goals/outcomes for autistic children. Across the studies, a clear pattern emerged: a move away from deficit-focused aims, such as reducing autistic traits, towards goals/outcomes centred on inclusion, autonomy, emotional well-being, diverse communication, and, to a lesser extent, sensory support. These shifts were evident in both quantitative and qualitative findings, with several recent studies explicitly rejecting normalisation-based goals/outcomes in favour of those that emphasise identity, agency, and quality of life (e.g. Gormley et al., 2024; Schuck et al., 2024). Rather than suggesting a complete paradigm transformation, the current evidence indicates an emerging and uneven but meaningful trend towards more neurodiversity-affirming priorities. These findings highlight important implications for future research and practice, the need to ensure that goal-setting processes meaningfully incorporate autistic perspectives, attend to environmental and relational contexts, and reflect outcomes that align with autistic children’s lived experiences and definitions of well-being. While this review alone cannot establish a definitive shift across the entire field, the pattern observed across the included studies suggests that more participatory, identity-affirming, and context-sensitive approaches may be required to align support goals with what autistic communities identify as meaningful.

Social inclusion emerged as an important developmental goal/outcome across the studies reviewed. In the results, three studies specifically highlighted community inclusion and social acceptance as key priorities (Clark & Adams, 2020; Pfeiffer et al., 2016; Schuck et al., 2024), emphasising the need for autistic children to be understood, accepted, and meaningfully included within their wider communities. Beyond this, several additional studies described related priorities centred on peer relationships, friendship, participation, and connectedness (Gormley et al., 2024; Laubscher et al., 2024; Lindsay et al., 2016; Petrina et al., 2015), illustrating that social inclusion is experienced not only at the community level but also within everyday interpersonal interactions. Taken together, these studies highlight that inclusion extends far beyond physical presence in shared spaces; it encompasses emotional safety, authentic relationships, and a sense of belonging. Such a holistic view stands in contrast to traditional behavioural frameworks that prioritise surface-level integration or compliance without ensuring genuine engagement or connection.

However, some tensions were also evident. Some educational frameworks continue to prioritise behavioural conformity, placing greater emphasis on outwardly observable behaviours rather than autistic children’s lived experiences and expressed preferences (Roberts & Simpson, 2016; Schuck et al., 2022). This enduring prioritisation may reflect entrenched educational paradigms that equate visible behavioural conformity with successful social integration, possibly due to systemic pressures or limited understanding of neurodiversity. Addressing this tension requires deliberate efforts to centre autistic perspectives in education policy and practice, ensuring that indicators of meaningful participation align with autistic-defined experiences of connection and belonging.

Communication goals/outcomes featured across seven studies, reflecting a shift towards valuing children’s preferred and functional methods of expression rather than prioritising spoken language alone (Brock et al., 2019; De Korte et al., 2022; DuBay et al., 2018; Gormley et al., 2024; Laubscher et al., 2024; Schuck et al., 2024; Waddington et al., 2024). As highlighted in the results, these studies recognised the importance of multimodal communication, such as AAC, sign language, and other non-verbal strategies, and the role of caregiver responsiveness in supporting children’s communicative autonomy. Schuck et al. (2024) further emphasised this direction, explicitly advocating for acceptance of diverse autistic communication methods and cautioning against interventions that privilege speech over equally valid forms of expression. At the same time, variability remained: some caregivers in DuBay et al. (2018) continued to prioritise spoken language, particularly for safety and community participation, and Brock et al. (2019) referenced communication broadly without specifying modality. Collectively, these findings illustrate an ongoing shift towards flexible, functional, and child-led communication approaches, while also underscoring the persistent ambiguity in how communication goals/outcomes are conceptualised across research and practice.

Child well-being emerged as a consistently prioritised goal/outcome across eight studies, reflecting a shift towards supporting autistic children’s emotional safety, regulation, and overall quality of life (Bent et al., 2024; Clark & Adams, 2020; De Korte et al., 2022; Gormley et al., 2024; Laubscher et al., 2024; Lindsay et al., 2016; Petrina et al., 2015; Sulek et al., 2024). As highlighted in the results, these studies emphasised aspects of well-being such as mental and emotional health, reduced stress, and strengthened regulatory support, indicating a movement away from behavioural compliance or isolated skill acquisition towards more holistic developmental priorities. Importantly, emerging scholarship underscores the need to centre autistic individuals’ own definitions of quality of life, which encompass sensory comfort, autonomy, identity affirmation, and meaningful relationships, dimensions often overlooked in traditional behavioural or health-based frameworks (Evers et al., 2022; Mason et al., 2018; McConachie et al., 2020). Together, these findings highlight the importance of support approaches that prioritise emotional safety, respect sensory and regulatory needs, and affirm autistic identity. Aligning goals/outcomes with autistic-affirmed understandings of well-being will require systemic change in how support effectiveness is conceptualised, measured, and prioritised.

Autonomy emerged as a central goal/outcome across six studies, reflecting a growing recognition of autistic children’s right to agency and self-direction (Bent et al., 2024; Gormley et al., 2024; Pfeiffer et al., 2016; Schuck et al., 2024; Waddington et al., 2023, 2024). As highlighted in the results, these studies emphasised supporting children to make independent choices, express preferences, and act as causal agents in their own lives, rather than relying on compliance-driven or prescriptive models. For example, Bent et al. (2024) prioritised enabling infant autonomy through responsive, adaptive environments, while Gormley et al. (2024) and Waddington et al. (2023, 2024) identified self-determination and authentic autistic identity as key developmental outcomes. Pfeiffer et al. (2016) and Schuck et al. (2024) further underscored autonomy’s importance, highlighting long-term independence, self-advocacy, and interdependence as integral components of autistic well-being. Collectively, these findings signal a broader shift towards support practices that recognise autonomy as foundational rather than optional, central to psychological well-being, identity development, and meaningful participation. Prioritising autonomy requires moving beyond paternalistic or compliance-based approaches and instead creating environments that honour children’s preferences, communication styles, and sensory needs, enabling them to thrive on their own terms.

Although many studies recognised sensory needs as critical for autistic children, explicit sensory-related goals/outcomes were prioritised in six studies (Bent et al., 2024; Laubscher et al., 2024; Schuck et al., 2024; Sulek et al., 2024; Waddington et al., 2023, 2024). Across these studies, participants highlighted the importance of sensory accommodations, environmental adaptations, and support for regulation as fundamental to autistic well-being. As the results demonstrated, however, sensory goals/outcomes were still less frequently prioritised compared to areas such as communication, autonomy, and well-being. Given the centrality of sensory regulation to autistic lived experience, this limited explicit prioritisation represents an important gap. Future work may benefit from incorporating sensory considerations more systematically, clearly defining goals/outcomes related to sensory comfort, accessibility, and emotional regulation.

While stakeholders broadly agreed on the importance of social inclusion, communication, and autonomy as key developmental goals/outcomes, notable differences emerged in how these were prioritised and defined. Professionals often emphasised social skills training and conventional communication benchmarks (Brock et al., 2019; Lindsay et al., 2016; Petrina et al., 2015; Waddington et al., 2023). These patterns may be influenced by long-standing educational expectations or developmental norms that emphasise observable social behaviours. These differences were particularly evident in relation to play while professionals prioritised structured play goals/outcomes, autistic individuals and their families expressed a preference for flexible, self-directed activities (Waddington et al., 2023).

More broadly, autistic participants and families tended to prioritise goals/outcomes that supported self-determination, environmental adaptation, emotional well-being, and overall quality of life (Pfeiffer et al., 2016; Schuck et al., 2024; Sulek et al., 2024; Waddington et al., 2023, 2024). These priorities often differed from those emphasised in studies where goals/outcomes focused more on behaviour reduction, academic performance, or conventional developmental expectations (e.g. Brock et al., 2019; Derguy et al., 2015). Such differences reflect the diversity of perspectives across stakeholder groups and highlight the value of considering how goals/outcomes align with what autistic individuals and their families define as meaningful. These findings underscore the potential benefits of goal-setting processes that incorporate autistic perspectives and support approaches that are responsive to individual preferences, needs, and lived experiences.

### Limitations

A key limitation of the literature reviewed is the predominance of studies conducted in Western contexts, which restricts insight into how diverse cultural values shape goal-setting practices and may limit the global applicability of findings. Cultural attitudes towards autism, disability, and family roles play a significant role in shaping developmental goals/outcomes, yet perspectives from non-Western contexts remain underrepresented. Even among Western samples, many studies relied on small, relatively homogeneous participant groups, often with limited representation of racially and ethnically diverse communities, lower-income families, or individuals without formal autism diagnoses. While some studies included non-binary and gender-diverse participants or spanned multiple countries, the overall lack of global and intersectional representation constrains the ability to generalise findings across different age groups, identities, and sociocultural settings. Although participatory approaches are becoming more common, several studies lacked meaningful involvement of autistic individuals in research design, evaluation, or interpretation (Clark & Adams, 2020; Lindsay et al., 2016). Even in studies with autistic participants, power dynamics and framing may have constrained the extent to which participant perspectives shaped the prioritisation of goals/outcomes.

This review has several methodological limitations that warrant consideration when interpreting the findings. First, by including only peer-reviewed literature published in English, we likely excluded valuable perspectives from non-English-speaking communities and grey literature. This limitation may have skewed the findings towards Western, individualistic cultural models of support and underrepresented collectivist or Indigenous frameworks that might prioritise different goals/outcomes.

Second, synthesising qualitative and quantitative studies posed interpretive challenges, particularly when studies used different terminologies or theoretical orientations. For instance, some studies conceptualised “independence” in behaviourist terms (e.g. task completion), while others framed it in relation to autonomy and agency. This lack of conceptual consistency may have led to subtle weighting towards studies with more explicit or measurable goal/outcome statements, particularly those using predefined outcome categories. While this variation introduced complexity, the overarching conclusions, particularly the shared prioritisation of communication, well-being, autonomy, and inclusion, remained consistent across methodological approaches.

Third, although we applied separate quality appraisal tools to qualitative and quantitative studies, this may have introduced some inconsistency in how rigour was assessed. However, we mitigated this by explicitly documenting our criteria and applying a neurodiversity-affirming lens to guide interpretation across all study types.

### Future Research

Future research would benefit from clearer conceptual definitions and greater transparency in how goals/outcomes are framed and measured across methodologies. More specifically, studies often use terms like “independence,” “communication,” or “social skills” without defining what these mean in context, leading to ambiguity about whether the goal reflects behavioural compliance, functional ability, or lived well-being. For instance, “independence” may be framed in one study as completing tasks without assistance, while another defines it as making autonomous choices with support. Future studies should explicitly define such terms and link them to theoretical or community-informed frameworks, specifying whether goals/outcomes are intended to reflect behavioural benchmarks, developmental milestones, or quality-of-life indicators. While large-scale, co-produced, cross-cultural research is ideal, it may not always be feasible. Smaller-scale studies can still contribute meaningfully by involving autistic people in goal/outcome setting, ensuring contextual and cultural reflexivity, and making explicit the values underlying chosen outcomes. To support synthesis across diverse designs, future reviews may benefit from using flexible meta-frameworks that allow for comparisons across different epistemological traditions while preserving complexity.

Future research should expand geographic and linguistic inclusion to ensure that goals/outcomes reflect the diversity of autistic communities worldwide. While some included studies involved participants from multiple countries (e.g. Bent et al., 2024; Schuck et al., 2024), most were conducted in Western, English-speaking contexts, with limited representation of Indigenous perspectives or non-Western conceptualisations of autism. In addition, only a few studies explicitly included gender-diverse participants or those without a formal autism diagnosis, despite growing recognition that these groups often face barriers to both diagnosis and appropriate support.

This gap matters because current support goals/outcomes may disproportionately reflect the values and norms of majority groups, overlooking priorities shaped by cultural, gendered, or diagnostic access experiences. Including non-binary individuals, multiply marginalised participants, and self-identified autistic people in future studies will help broaden and validate the spectrum of priorities that matter to autistic communities.

To develop genuinely neurodiversity-affirming goals/outcomes, future studies must not only prioritise co-design methodologies that centre autistic voices across all stages, from shaping research questions to analysing findings and forming recommendations, but also address a critical gap exposed by this review, the lack of appropriate measurement tools. At present, we have few validated instruments capable of capturing outcomes that are meaningful to autistic individuals and their families, or that reflect principles like autonomy, inclusion, sensory comfort, and environmental fit. Without such tools, researchers and practitioners are limited in their ability to design, deliver, or evaluate neurodiversity-affirming supports. This is a pressing issue for the field. While large-scale co-produced, cross-cultural projects may not always be feasible, smaller-scale studies can still make meaningful contributions by involving local autistic advisors, using inclusive recruitment strategies, and explicitly reflecting on whose voices are included or excluded. These practical steps, alongside the development of new, community-informed assessment tools, can shift goal setting from externally imposed frameworks to ones grounded in lived experience, ensuring that both research and practice are truly aligned with what matters to autistic people.

## Supplemental Material

sj-docx-1-aut-10.1177_13623613261433132 – Supplemental material for Priorities and Perspectives Regarding Goals and Outcomes of Support for Autistic Children Under 12 Years: A Systematic Review Supplemental material, sj-docx-1-aut-10.1177_13623613261433132 for Priorities and Perspectives Regarding Goals and Outcomes of Support for Autistic Children Under 12 Years: A Systematic Review by Phoebe Jordan, Hannah Waddington, Matt Hammond, Mirko Uljarevic, Willow J Sainsbury and Jessica Tupou in Autism

sj-docx-2-aut-10.1177_13623613261433132 – Supplemental material for Priorities and Perspectives Regarding Goals and Outcomes of Support for Autistic Children Under 12 Years: A Systematic Review Supplemental material, sj-docx-2-aut-10.1177_13623613261433132 for Priorities and Perspectives Regarding Goals and Outcomes of Support for Autistic Children Under 12 Years: A Systematic Review by Phoebe Jordan, Hannah Waddington, Matt Hammond, Mirko Uljarevic, Willow J Sainsbury and Jessica Tupou in Autism

sj-docx-3-aut-10.1177_13623613261433132 – Supplemental material for Priorities and Perspectives Regarding Goals and Outcomes of Support for Autistic Children Under 12 Years: A Systematic Review Supplemental material, sj-docx-3-aut-10.1177_13623613261433132 for Priorities and Perspectives Regarding Goals and Outcomes of Support for Autistic Children Under 12 Years: A Systematic Review by Phoebe Jordan, Hannah Waddington, Matt Hammond, Mirko Uljarevic, Willow J Sainsbury and Jessica Tupou in Autism

sj-docx-4-aut-10.1177_13623613261433132 – Supplemental material for Priorities and Perspectives Regarding Goals and Outcomes of Support for Autistic Children Under 12 Years: A Systematic Review Supplemental material, sj-docx-4-aut-10.1177_13623613261433132 for Priorities and Perspectives Regarding Goals and Outcomes of Support for Autistic Children Under 12 Years: A Systematic Review by Phoebe Jordan, Hannah Waddington, Matt Hammond, Mirko Uljarevic, Willow J Sainsbury and Jessica Tupou in Autism

sj-docx-5-aut-10.1177_13623613261433132 – Supplemental material for Priorities and Perspectives Regarding Goals and Outcomes of Support for Autistic Children Under 12 Years: A Systematic Review Supplemental material, sj-docx-5-aut-10.1177_13623613261433132 for Priorities and Perspectives Regarding Goals and Outcomes of Support for Autistic Children Under 12 Years: A Systematic Review by Phoebe Jordan, Hannah Waddington, Matt Hammond, Mirko Uljarevic, Willow J Sainsbury and Jessica Tupou in Autism

sj-docx-6-aut-10.1177_13623613261433132 – Supplemental material for Priorities and Perspectives Regarding Goals and Outcomes of Support for Autistic Children Under 12 Years: A Systematic Review Supplemental material, sj-docx-6-aut-10.1177_13623613261433132 for Priorities and Perspectives Regarding Goals and Outcomes of Support for Autistic Children Under 12 Years: A Systematic Review by Phoebe Jordan, Hannah Waddington, Matt Hammond, Mirko Uljarevic, Willow J Sainsbury and Jessica Tupou in Autism

sj-docx-7-aut-10.1177_13623613261433132 – Supplemental material for Priorities and Perspectives Regarding Goals and Outcomes of Support for Autistic Children Under 12 Years: A Systematic Review Supplemental material, sj-docx-7-aut-10.1177_13623613261433132 for Priorities and Perspectives Regarding Goals and Outcomes of Support for Autistic Children Under 12 Years: A Systematic Review by Phoebe Jordan, Hannah Waddington, Matt Hammond, Mirko Uljarevic, Willow J Sainsbury and Jessica Tupou in Autism

sj-docx-8-aut-10.1177_13623613261433132 – Supplemental material for Priorities and Perspectives Regarding Goals and Outcomes of Support for Autistic Children Under 12 Years: A Systematic Review Supplemental material, sj-docx-8-aut-10.1177_13623613261433132 for Priorities and Perspectives Regarding Goals and Outcomes of Support for Autistic Children Under 12 Years: A Systematic Review by Phoebe Jordan, Hannah Waddington, Matt Hammond, Mirko Uljarevic, Willow J Sainsbury and Jessica Tupou in Autism

sj-docx-9-aut-10.1177_13623613261433132 – Supplemental material for Priorities and Perspectives Regarding Goals and Outcomes of Support for Autistic Children Under 12 Years: A Systematic Review Supplemental material, sj-docx-9-aut-10.1177_13623613261433132 for Priorities and Perspectives Regarding Goals and Outcomes of Support for Autistic Children Under 12 Years: A Systematic Review by Phoebe Jordan, Hannah Waddington, Matt Hammond, Mirko Uljarevic, Willow J Sainsbury and Jessica Tupou in Autism
