# Supplementary material for: Priorities and Perspectives Regarding Goals and Outcomes of Support for Autistic Children Under 12 Years: A Systematic Review
Source: Autism. 2026 Apr 20;30(6):1416–29. doi: 10.1177/13623613261433132 (PMC13187217; doi:10.1177/13623613261433132)
Supplement: sj-docx-2-aut-10.1177_13623613261433132 – Supplemental material for Priorities and Perspectives Regarding Goals and Outcomes of Support for Autistic Children Under 12 Years: A Systematic Review [file sj-docx-2-aut-10.1177_13623613261433132.docx]

Supplementary Materials 2.

*Neurodiversity-Affirming Framework for Evaluating Studies*

**Neurodiversity-Affirming Framework for Evaluating Studies**

To evaluate the extent to which each study’s conceptualisation, design, and procedures align with neurodiversity-affirming principles, a structured framework was developed. This approach offers a systematic and consistent way to assess whether studies uphold affirming and respectful practices for autistic people. The five core principles included in this framework are: (a) a strengths-based focus, (b) self-determination and autonomy, (c) adaptation of the environment and upskilling of others, (d) diverse communication methods, and (e) respect for sensory and processing differences. These principles are not arbitrary; each contributes to key aspects of quality of life for autistic individuals. While quality of life is highly individual and shaped by many idiosyncratic factors, these principles reflect widely documented priorities identified by autistic people themselves.

All evaluations are based on how each study’s authors have conceptualised, designed, and conducted their research, including decisions around target goals, procedures, and outcomes. Evaluations are not based on the actions or choices of participants within the study. That is, a study could still meet the criteria even if participants demonstrate behaviours or express attitudes that do not align with one or more of the five core neurodiversity-affirming principles.

This framework assesses studies based on the presence or absence of five core principles, with each principle classified as:

- Yes – The principle is discussed in a way that is neurodiversity affirming in both the study’s conceptualisation and conduct.
- No – The principle is discussed in a way that is not neurodiversity affirming in either the study’s conceptualisation or conduct.

A score of N/A may be given for Principle 4 (Diverse Communication Methods) and Principle 5 (Respect for Sensory and Processing Differences) if that principle was not discussed at all in the article.

Data to inform decisions can be drawn from how the study is conceptualised in the Introduction and described in the Methodology, including study design, goals, procedures, and outcomes.

1. **Strengths-Based Focus**

Definition:

A strengths-based approach emphasizes the abilities, interests, and potential of autistic people rather than focusing on deficits or impairments (Leinfuss & O’Hara, 2024).

Evaluation Criteria:

Studies were coded "Yes" only if a strengths-based conceptualisation of autism was clearly evident throughout the study. This includes studies that explicitly centred autistic strengths (e.g., pattern recognition, deep focus, creativity) and used them to inform the conceptualisation, goals, procedures, and outcomes of the study.

Studies were coded as “No” if they framed autism in deficit terms or failed to apply a strengths-based lens consistently across the study. If a study began with a medical or deficit-based definition of autism but clearly adopted a neurodiversity-affirming perspective in its conceptualisation, goals, procedures, and interpretation of findings, it was not automatically excluded on that basis alone. In such cases, the overall application of a strengths-based approach throughout the study was used to inform coding decisions.

1. **Self-Determination and Autonomy**

Definition:

Self-determination involves enabling autistic people to have agency over their own lives, make choices, and express preferences in ways that align with their strengths and abilities (Ryan et al., 2024; Webster et al., 2022).

Evaluation Criteria:

Studies were coded "Yes" only if they centred autistic voices—meaning that the research was co-designed with autistic people or meaningfully incorporated autistic input into the conceptualisation of the research. In addition, these studies needed to emphasise individual preferences or explicitly promote autonomy and self-advocacy in the design, goals, procedures, and outcomes.

Studies were coded "No" if they failed to demonstrate a consistent commitment to self-determination and autonomy.

1. **Adaptation of the Environment and Upskilling of People**

Definition:

A neurodiversity-affirming approach prioritises modifying environments, upskilling people, and adjusting expectations rather than attempting to “normalise” the autistic individual (Waddington et al., 2023). This includes changing the physical, social, and communicative context to better meet autistic needs, rather than focusing on changing autistic traits (Waddington et al., 2023).

Evaluation Criteria:

Studies were coded “Yes” only if the authors demonstrated a clear commitment to the principle of adapting the environment and/or upskilling the people around the autistic individual (e.g., parents, teachers, therapists). This includes how the study conceptualises support needs—not solely as individual deficits to be ‘treated,’ but as contextual or relational challenges that can be addressed through environmental changes or shifts in others’ knowledge, expectations, or behaviours. While examples such as inclusive education practices, sensory-friendly modifications, or adult training may be present in some support-focused studies, the core criterion is whether the study frames change as something that can, and should, occur around the autistic person, not only within them.

If a study focused on altering autistic traits (e.g., enforcing eye contact, suppressing stimming) or failed to conceptualise support needs in a way that acknowledged the role of environmental or relational adaptation, it was coded as “No.”

1. **Diverse Communication Methods**

Definition:

Neurodiversity-affirming approaches recognize that communication is varied, and autistic people may use AAC (Augmentative and Alternative Communication), sign language, echolalia or other non-traditional means (Gaddy & Crow, 2023).

Evaluation Criteria:

Studies were coded “Yes” only if diverse communication methods were acknowledged, respected, and actively supported throughout the study’s conceptualisation, design, goals, procedures, and outcomes. This includes explicit validation of non-speech communication and a clear rejection of speech-only norms.

A "No" was assigned if the study prioritised spoken language exclusively, discouraged or attempted to replace non-speech communication or failed to provide meaningful accommodation for varied communication needs, even if alternative communication was mentioned in passing.

Studies were coded "N/A" if communication was not addressed in any way within the study.

1. **Respect for Sensory and Processing Differences**

Definition:

Autistic people often experience unique sensory processing patterns. Respecting these differences involves creating supportive environments that accommodate sensory needs, rather than attempting to suppress, reduce, or “normalise” sensory responses (Kadlaskar et al., 2022; Morgan, 2019; Sibeoni et al., 2022).

Evaluation Criteria:

Studies were coded "Yes" only if respect for sensory and processing differences was embedded throughout the study’s conceptualisation, design, goals, procedures, and outcomes. This includes incorporating sensory-friendly adaptations (e.g., flexible lighting, reduced noise, access to sensory tools or materials) and showing a clear commitment to accommodating sensory needs in both the environment and the overall approach.

Studies were coded "No" if they attempted to reduce or suppress sensory differences without clear evidence that those differences were causing harm to the individual or others, or if such efforts were made without the informed consent of the individual. A “No” rating was also applied if the study framed sensory differences as problems to be fixed rather than needs to be supported, or if sensory needs were mentioned but not meaningfully accommodated within the study’s design or implementation.

A study that simply failed to mention sensory or processing differences at all was coded “N/A”, not “No.” A “No” rating was reserved for studies that acknowledged sensory differences but failed to respect or accommodate them meaningfully.
