# Supplementary material for: Priorities and Perspectives Regarding Goals and Outcomes of Support for Autistic Children Under 12 Years: A Systematic Review
Source: Autism. 2026 Apr 20;30(6):1416–29. doi: 10.1177/13623613261433132 (PMC13187217; doi:10.1177/13623613261433132)
Supplement: sj-docx-3-aut-10.1177_13623613261433132 – Supplemental material for Priorities and Perspectives Regarding Goals and Outcomes of Support for Autistic Children Under 12 Years: A Systematic Review [file sj-docx-3-aut-10.1177_13623613261433132.docx]

Supplementary Table 3.

*Full-Text Articles Excluded and Reasons*

| **Author(s), Year** | **Title** | **Reason for Exclusion** |
| --- | --- | --- |
| Blaine 2023 | Priorities, barriers, and facilitators for nutrition-related care for autistic children: a qualitative study comparing interdisciplinary health professional and parent perspectives | Wrong patient population |
| Burrell 2017 | The Experiences of Fathers Who Have Offspring with Autism Spectrum Disorder. | Wrong patient population |
| Carbone 2013 | Parent and Pediatrician Perspectives Regarding the Primary Care of Children with Autism Spectrum Disorders | Outside of publication timeframe |
| Cheak‐Zamora 2015 | 'Transitions are Scary for our Kids, and They're Scary for us': Family Member and Youth Perspectives on the Challenges of Transitioning to Adulthood with Autism. | Wrong patient population |
| Clark 2020 | Parent and Teacher Ratings of Social Skills, Peer Play and Problem Behaviours in Children with Autism Spectrum Disorder. | Does not focus on goals/outcomes |
| Damiao 2024 | Parent Perspectives on Assisted Communication and Autism Spectrum Disorder. | Wrong patient population |
| Dwyer 2024 | Community views of neurodiversity, models of disability and autism intervention: Mixed methods reveal shared goals and key tensions | Does not focus on goals/outcomes |
| Edwards 2016 | “More than blowing bubbles”: What parents want from therapists working with children with autism spectrum disorder. | Does not focus on goals/outcomes |
| Edwards 2017 | The early intervention message: perspectives of parents of children with autism spectrum disorder. | Does not focus on goals/outcomes |
| Ekas 2019 | Religiosity/Spirituality and Mental Health Outcomes in Mothers of Children with Autism Spectrum Disorder: The Mediating Role of Positive Thinking. | Does not focus on goals/outcomes |
| EsquilínNieves 2023 | Examining Perspectives of Latina Mothers and Speech-Language Pathologists Through Dyadic Interviews: Implications for Latine Children on the Autism Spectrum. | Wrong patient population |
| Fletcher-Watson 2016 | Attitudes of the autism community to early autism research | Does not focus on goals/outcomes |
| Genc-Tosun 2017 | Teaching multi-step requesting to children with autism spectrum disorder using systematic instruction and a speech-generating device. | Does not focus on goals/outcomes |
| Gevarter 2022 | Naturalistic Communication Training for Early Intervention Providers and Latinx Parents of Children with Signs of Autism. | Does not focus on goals/outcomes |
| Hatfield 2023 | "Stop and just breathe for a minute": perspectives of children on the Autism Spectrum and their caregivers on a Mindfulness Group. | Wrong patient population |
| Hersh 2024 | Community Member Views on Autism Intervention: Effects of Closeness to Autistic People with Intellectual Disabilities And Nonspeaking Autistic People | Wrong patient population |
| Leroux 2024 | Views of Parents on Supporting Language and Literacy for Their Children With Complex Communication Needs. | Wrong patient population |
| London 2020 | Animal Assisted Therapy for Children and Adolescents with Autism Spectrum Disorder: Parent perspectives. | Wrong patient population |
| Manohar 2019 | Brief Parent-Mediated Intervention for Children with Autism Spectrum Disorder: A Feasibility Study from South India. | Does not focus on goals/outcomes |
| Milgramm 2023 | Brief Report: Family Recreation for Individuals with Autism Spectrum Disorder. | Wrong patient population |
| Parker 2020 | Exploring Exceptions and Discovering Solutions: A Case Presentation of Autism and the Family. | Does not focus on goals/outcomes |
| Parsons 2019 | Appropriateness of the TOBY Application, an iPad Intervention for Children with Autism Spectrum Disorder: A Thematic Approach. | Does not focus on goals/outcomes |
| Robert 2015 | When satisfaction is not directly related to the support services received: understanding parents' varied experiences with specialised services for children with developmental disabilities. | Wrong patient population |
| Rodger 2004 | Early Intervention for Children with Autism: Parental Priorities | Outside of publication timeframe |
| Román-Oyola 2018 | Play, Playfulness, and Self-Efficacy: Parental Experiences with Children on the Autism Spectrum. | Does not focus on goals/outcomes |
| Sandham 2022 | The perspectives of Australian speech pathologists in providing evidence‐based practices to children with autism. | Does not focus on goals/outcomes |
| Secora 2023 | Speech-Language Pathologists' Implementation of Theory of Mind Concepts Within Therapy: An Exploratory Survey. | Does not focus on autism |
| Sturrock 2023 | The impact of subtle language and communication difficulties on the daily lives of autistic children without intellectual disability: Parent perspectives. | Wrong patient population |
| Tesfaye 2022 | Autism voices: Perspectives of the needs, challenges, and hopes for the future of autistic youth | Wrong patient population |
| Thompson 2017 | Long-Term Perspectives of Family Quality of Life Following Music Therapy With Young Children on the Autism Spectrum: A Phenomenological Study. | Does not focus on goals/outcomes |
| Tucker 2013 | Parents’ Perspectives of Collaboration with School Professionals: Barriers and Facilitators to Successful Partnerships in Planning for Students with ASD | Outside of publication timeframe |
