# Supplementary material for: Priorities and Perspectives Regarding Goals and Outcomes of Support for Autistic Children Under 12 Years: A Systematic Review
Source: Autism. 2026 Apr 20;30(6):1416–29. doi: 10.1177/13623613261433132 (PMC13187217; doi:10.1177/13623613261433132)
Supplement: sj-docx-4-aut-10.1177_13623613261433132 – Supplemental material for Priorities and Perspectives Regarding Goals and Outcomes of Support for Autistic Children Under 12 Years: A Systematic Review [file sj-docx-4-aut-10.1177_13623613261433132.docx]

**Supplementary Materials 4.**

*Additional Study and Participant characteristics*

| Author(s) and year | Geographical location | Gender | Ethnicity | Further details about role/identity |
| --- | --- | --- | --- | --- |
| Bent et al., 2024 | Australia, United States, United Kingdom, India | 74% female  21% male  5% non-binary | NS | 238 participants: 128 autistic and 110 non-autistic individuals, including parents, professionals, and researchers. Many held intersecting roles (e.g., autistic parent, professional researcher).  Participants were recruited through social media and snowball sampling. |
| Brock et al., 2019 | United States | NS | NS | 99 teachers (70 special education teachers, 29 general education teachers) of autistic students.  Participants were recruited through emails sent to principals of schools with survey links. |
| Clark & Adams 2020 | Australia | Phase 1: 98.5% female, 1.5% male  Phase 2: 88.9% female, 11.1% male | NS | All participants were parents of school-aged autistic children.  Participants were recruited through social media and snowball sampling. |
| De Korte et al., 2022 | Netherlands | 71% female  29% male  100% of children were male | NS | Participants were parents of young autistic children (aged 3–7 years) who had taken part in a 14-week PRT parent group training program.  Participants were recruited through an email or phone call after they completed the PRT-PG intervention. |
| Derguy et al., 2015 | France | 55.1% female  44.9% male | NS | 78 parents of autistic children aged 3-10 years.  Participants were recruited from five centres specialising in autism diagnoses, seven parent associations, five centres providing care to children and two schools. An information letter and consent form were sent by health and education professionals. |
| DuBay et al., 2018 | United States | LSS caregivers: 14 of 20 interviews were with mothers alone; the rest included combinations with husbands, aunts, grandmothers, or friends.  NLW caregivers: all but one were mothers. | 28 LSS  27 NLW | All participants were parents or primary caregivers of young children (aged 1–6 years) diagnosed as or suspected to be autistic.  Participants were recruited through a state chapter of a national ASD advocacy organisation through support group meetings, parent workshops, community events and membership networks. |
| Gormley et al., 2024 | Republic of Ireland and Northern Ireland | Autistic educators: 6 women, 3 men, 1 non-binary.  Autistic non-educators: 17 women, 7 men, 8 non-binary, 2 prefer not to say.  Non-autistic educators: 60 women, 5 men. | Autistic educators: 100% White  Autistic non-educators: 30 White, 3 Mixed race, 1 not reported.  Non-autistic educators: 63 White, 2 not reported. | 34 autistic non-educators  65 non-autistic educators  10 autistic educators  Participants were recruited through convenience and snowball sampling through recruitment flyers and social media channels. |
| Laubscher et al., 2024 | United States | 6 males  1 female | 5 participants identified as White  1 participant identified as both White and Black/African American  2 participants did not report their race | 6 participants were parents, 2 participants were grandparents who had assumed parenting responsibilities for their grandson and all participants spoke English.  Participants were recruited through emails to professionals, personal contacts, and organizations serving individuals on the autism spectrum and their families; and through social media. |
| Lindsay et al., 2016 | United Kingdom | 93% mothers  7% fathers | NS | Parents of 129 school-aged children with either language impairment (LI; *n* = 76) or who are autistic (autism; *n* = 53); recruited through mainstream schools as part of a larger prospective longitudinal study.  Participants were recruited through a prospective longitudinal study from mainstream primary and secondary schools. |
| Petrina et al., 2015 | Australia | 65 mothers  7 fathers  2 grandparents | NS | 74 caregivers of autistic children aged 6.4–10.4 years.  Participants were recruited through two organisations in New South Wales and Southern Australia. |
| Pfeiffer et al., 2016 | United States | Among autistic adults: 3 females, 2 males.  Parents' gender NS. | NS | Participants were 5 autistic adults and 5 autistic children. The autistic adults were aged 28–60, verbal communicators and members of support groups. The autistic children of the parent participants were aged 4–8, all diagnosed autistic and attending ABA classrooms with additional OT and speech therapy.  Parent participants were recruited through schools programs for children with ASD and through private therapy practices. Adult participants were recruited through adult ASD support groups and an ASD organisation providing resources and support for adults diagnosed with ASD. |
| Schuck et al., 2024 | United States, Canada, United Kingdon, Australia and Scandinavian countries. | 50.5% female  23.8% gender diverse  17.3% male  8.4% NS | 84.1% White  2.8% Mixed Race  2.8% Hispanic  1.9% Asian  1.4% Native American  0.9% Black  6.1% NS | 75.2% clinically diagnosed autistic people and 24.8% self-identified autistic people. The average age was 34.86 years. 29% received behavioural support as children, 49.1% did not and 19.2% were unsure.  Participants were recruited recruited online through social networking sites (e.g., Facebook, Twitter, Reddit) and by reaching out to autism organizations to send the advertisement to their listservs. |
| Sulek et al., 2024 | Australia and New Zealand | 82.9% female  12.7% male  4.4% non-binary  0.6% other | European Australian (73.5%)  New Zealand European (13.8%)  Māori (1.7%)  Aboriginal (0.6%)  Chinese (2.8%)  Indian (1.7%)  Other (8.8%) | 43.6% parents, 29.3% practitioners, 27.1% autistic adults. Some individuals identified with more than one role.  Participants were recruited via several mechanisms including: survey flyers shared, with permission, in social media groups whose membership represented our target stakeholders (i.e., autistic individuals, practitioners, parents of autistic children); recruitment calls by autism specific organisations to their members via social media or mailing lists; distribution of survey flyers and recruitment emails via the study authors’ professional networks and personal social media pages (e.g., LinkedIn, X, Facebook). |
| Waddington et al., 2023 | New Zealand and Australia | 83% female  9% male  8% gender diverse | NZ/Australian European: 79%  Māori: 8%  Asian: 12% | 41 autistic adults, 50 parents, 31 professionals; participants could hold multiple roles.  Participants were recruited through online autism-specific organisations in New Zealand and Australia and autism support groups for parents and autistic adults on Facebook. |
| Waddington et al., 2024 | New Zealand and Australia | 83% female  12% male  5% gender diverse | NZ/Australian European: 71%  Māori: 11%  Asian: 6%  Pacific Islander: 2%  Other: 11% | 87 autistic adults, 159 parents of autistic children, 80 professionals; participants could hold multiple roles.  Participants were recruited through online through autism support organisations and social media pages for autism support groups for autistic adults, parents and/or professionals in New Zealand and Australia. Snow ball sampling was used, as participants were invited to share the survey link with others. |

*Note*: Latino Spanish-speaking (LSS)

Non-Latino White (NLW)

Not specified (NS)
