# Supplementary material for: Priorities and Perspectives Regarding Goals and Outcomes of Support for Autistic Children Under 12 Years: A Systematic Review
Source: Autism. 2026 Apr 20;30(6):1416–29. doi: 10.1177/13623613261433132 (PMC13187217; doi:10.1177/13623613261433132)
Supplement: sj-docx-5-aut-10.1177_13623613261433132 – Supplemental material for Priorities and Perspectives Regarding Goals and Outcomes of Support for Autistic Children Under 12 Years: A Systematic Review [file sj-docx-5-aut-10.1177_13623613261433132.docx]

**Supplementary Materials 5.**

*JBI item and total scores using analytical cross-sectional checklist*

|  | 1. Criteria for inclusion in the sample clearly defined | 2. Study subjects and the setting described in detail | 3. Exposure measured in a valid and reliable way | 4. Objective, standard criteria used for measurement of the condition | 5. Confounding factors identified | 6. Strategies to deal with confounding factors stated | 7. Outcomes measured in a valid and reliable way | 8. Appropriate statistical analysis used | Total |
| --- | --- | --- | --- | --- | --- | --- | --- | --- | --- |
| Bent et al., 2024 | ✓ | ✓ | ✓ | × | ✓ | × | × | ✓ | 6/8 |
| Brock et al., 2019 | ✓ | ✓ | ✓ | ✓ | × | × | ✓ | ✓ | 6/8 |
| Gormley et al., 2024 | ✓ | ✓ | ✓ | ✓ | × | × | ✓ | ✓ | 6/8 |
| Lindsay et al., 2016 | ✓ | ✓ | ✓ | ✓ | × | × | ✓ | ✓ | 6/8 |
| Petrina et al., 2015 | ✓ | ✓ | ✓ | ✓ | ✓ | × | ✓ | ✓ | 7/8 |
| Sulek et al., 2024 | ✓ | ✓ | ✓ | ✓ | ✓ | ✓ | ✓ | ✓ | 8/8 |
| Waddington et al., 2024 | ✓ | ✓ | ✓ | ✓ | ✓ | ✓ | ✓ | ✓ | 8/8 |
