# Supplementary material for: Priorities and Perspectives Regarding Goals and Outcomes of Support for Autistic Children Under 12 Years: A Systematic Review
Source: Autism. 2026 Apr 20;30(6):1416–29. doi: 10.1177/13623613261433132 (PMC13187217; doi:10.1177/13623613261433132)
Supplement: sj-docx-6-aut-10.1177_13623613261433132 – Supplemental material for Priorities and Perspectives Regarding Goals and Outcomes of Support for Autistic Children Under 12 Years: A Systematic Review [file sj-docx-6-aut-10.1177_13623613261433132.docx]

**Supplementary Materials 6.**

*JBI item and total scores using qualitative research checklist*

|  | 1. Congruity between stated philosophical perspective and the research methodology | 2. Congruity between research methodology and research question or objectives | 3. Congruity between research methodology and methods used to collect data | 4. Congruity between research methodology and representation and analysis of data | 5. Congruity between research methodology and interpretation of results | 6. Statement locating researcher culturally or theoretically | 7. Influence of researcher on research, and vice- versa | 8. Participants, and their voices, adequately represented | 9. Research ethical | 10. Conclusions drawn in the research report flow from the analysis, or interpretation, of the data | Total |
| --- | --- | --- | --- | --- | --- | --- | --- | --- | --- | --- | --- |
| Clark & Adams, 2020 | ✓ | ✓ | ✓ | ✓ | ✓ | × | × | ✓ | ✓ | ✓ | 8/10 |
| De Korte et al., 2022 | ✓ | ✓ | ✓ | ✓ | ✓ | ✓ | ✓ | ✓ | ✓ | ✓ | 10/10 |
| Derguy et al., 2015 | ✓ | ✓ | ✓ | ✓ | ✓ | × | ✓ | ✓ | ✓ | ✓ | 9/10 |
| DuBay et al., 2018 | × | ✓ | ✓ | ✓ | ✓ | × | × | ✓ | ✓ | ✓ | 7/10 |
| Laubscher et al., 2014 | ✓ | ✓ | ✓ | ✓ | ✓ | × | × | ✓ | ✓ | ✓ | 8/10 |
| Pfeiffer et al. 2016 | ✓ | ✓ | ✓ | ✓ | ✓ | ✓ | ✓ | ✓ | ✓ | ✓ | 10/10 |
| Schuck et al., 2024 | ✓ | ✓ | ✓ | ✓ | ✓ | ✓ | ✓ | ✓ | ✓ | ✓ | 10/10 |
| Waddington et al., 2023 | ✓ | ✓ | ✓ | ✓ | ✓ | ✓ | ✓ | ✓ | ✓ | ✓ | 10/10 |
