# Supplementary material for: Priorities and Perspectives Regarding Goals and Outcomes of Support for Autistic Children Under 12 Years: A Systematic Review
Source: Autism. 2026 Apr 20;30(6):1416–29. doi: 10.1177/13623613261433132 (PMC13187217; doi:10.1177/13623613261433132)
Supplement: sj-docx-7-aut-10.1177_13623613261433132 – Supplemental material for Priorities and Perspectives Regarding Goals and Outcomes of Support for Autistic Children Under 12 Years: A Systematic Review [file sj-docx-7-aut-10.1177_13623613261433132.docx]

**Supplementary Materials 7.**

*Differences and alignment in perspectives across participant groups*

| Author(s) and year | Differences and alignment in perspectives across participant groups |
| --- | --- |
| Bent et al., 2024 | Autistic adults and non-autistic participants largely agreed on the need for parental education and environmental supports, but some parents and professionals supported early labelling. |
| Brock et al., 2019 | Teachers and researchers agreed on the need for better implementation of EBPs, but teachers prioritized academic outcomes while research emphasized communication. |
| Clark & Adams 2020 | Only one participant group. |
| Derguy et al., 2015 | Only one participant group. |
| DuBay et al., 2018 | Caregivers prioritized verbal communication and structured support, while professionals lacked cultural competence and bilingual resources. |
| Gormley et al., 2024 | Broad agreement across educators and autistic individuals, with academic goals deprioritized. |
| Laubscher et al., 2024 | Parents broadly supported AAC but faced barriers from professionals when advocating for flexible, multimodal communication. |
| Lindsay et al., 2016 | Parents were generally satisfied with their role in decision-making but had different priorities, autistic children’s parents emphasized peer relationships, while LI parents focused on literacy. |
| Manon et al., 2022 | Only one participant group. |
| Petrina et al., 2015 | Only one participant group. |
| Pfeiffer et al., 2016 | Parents emphasized long-term security and structured planning, while autistic adults valued autonomy and self-determined support. |
| Schuck et al., 2024 | Only one participant group. |
| Sulek et al., 2024 | Autistic adults and professionals aligned in rejecting goals focused on reducing or ‘normalising’ core autistic traits, such as sensory seeking behaviours, focused interests, and neurodivergent play styles, whereas parents were more open to these behaviour-change goals, potentially reflecting real-world concerns about their child’s participation and acceptance in everyday environments. |
| Waddington et al., 2023 | All groups agreed on quality of life and autonomy, though professionals emphasized play skills more than autistic adults. There was consensus on prioritising well-being and autonomy over traditional support goals. |
| Waddington et al., 2024 | Autistic adults rated play and participation goals as less appropriate compared to parents and professionals. All groups emphasized the importance of quality of life and well-being over traditional support goals. |
