# Supplementary material for: Priorities and Perspectives Regarding Goals and Outcomes of Support for Autistic Children Under 12 Years: A Systematic Review
Source: Autism. 2026 Apr 20;30(6):1416–29. doi: 10.1177/13623613261433132 (PMC13187217; doi:10.1177/13623613261433132)
Supplement: sj-docx-8-aut-10.1177_13623613261433132 – Supplemental material for Priorities and Perspectives Regarding Goals and Outcomes of Support for Autistic Children Under 12 Years: A Systematic Review [file sj-docx-8-aut-10.1177_13623613261433132.docx]

**Supplementary Materials 8.**

*Alignment with neurodiversity-affirming principles*

| Author(s) and years |  | Alignment with neurodiversity-affirming principles | | | | | |  | |
| --- | --- | --- | --- | --- | --- | --- | --- | --- | --- |
|  |  | Strengths-based focus | Self-determination and autonomy | Adapt environments rather than child | Diverse communication methods | Respects sensory and processing differences |  | |  |
| Bent et al., 2024 |  | Yes | Yes | Yes | Yes | Yes |  | |  |
| Brock et al., 2019 |  | No | No | No | N/A | N/A |  | |  |
| Clark & Adams 2020 |  | Yes | No | No | N/A | N/A |  | |  |
| Derguy et al., 2015 |  | No | No | No | N/A | N/A |  | |  |
| De Korte et al., 2022 |  | No | No | No | No | N/A |  | |  |
| DuBay et al., 2018 |  | Yes | No | Yes | No | N/A |  | |  |
| Gormley et al., 2024 |  | Yes | Yes | Yes | Yes | Yes |  | |  |
| Laubscher et al., 2024 |  | Yes | No | Yes | Yes | Yes |  | |  |
| Lindsay et al., 2016 |  | No | No | No | N/A | N/A |  | |  |
| Petrina et al., 2015 |  | No | No | No | N/A | N/A |  | |  |
| Pfeiffer et al., 2016 |  | Yes | Yes | Yes | No | N/A |  | |  |
| Schuck et al., 2024 |  | Yes | Yes | Yes | N/A | Yes |  | |  |
| Sulek et al., 2024 |  | Yes | Yes | Yes | N/A | Yes |  | |  |
| Waddington et al., 2023 |  | Yes | Yes | Yes | Yes | Yes |  | |  |
| Waddington et al., 2024 |  | Yes | Yes | Yes | Yes | Yes |  | |  |
