# Supplementary material for: Priorities and Perspectives Regarding Goals and Outcomes of Support for Autistic Children Under 12 Years: A Systematic Review
Source: Autism. 2026 Apr 20;30(6):1416–29. doi: 10.1177/13623613261433132 (PMC13187217; doi:10.1177/13623613261433132)
Supplement: sj-docx-9-aut-10.1177_13623613261433132 – Supplemental material for Priorities and Perspectives Regarding Goals and Outcomes of Support for Autistic Children Under 12 Years: A Systematic Review [file sj-docx-9-aut-10.1177_13623613261433132.docx]

**Supplementary Materials 9.**

*Summary of goals/outcomes/ priorities*

| Author(s) and year | Findings |
| --- | --- |
| Bent et al., 2024 | Participants ranked goals on their acceptability (percentage indicates participants who agreed that the goal was acceptable). The following were identified as highly acceptable goals   - Helping caregivers understand and support infant’s feelings, needs, and behaviour (91%) - Improving infant’s quality of life (87%) - Respecting and enabling infant autonomy (85%) - Adapting the environment to meet the infant’s needs (83%) - Encouraging mutually enjoyable, warm parent-infant interactions (81%) - Parent education on autism and neurodivergence (79%)   The following were identified as less acceptable goals:   - Preventing autism (20%) - Reducing repetitive or autistic behaviours (28%) - Encouraging eye contact (34%)​ |
| Brock et al., 2019 | Participants identified their highest-priority goals for autistic students (percentage indicates participants who selected the goal as their single highest-priority). The goal priorities were as follow:   - Preacademic/academic skills (24.7%) - Social outcomes (18.0%) - Communication (17.4%) - Challenging behaviour (16.9%) - Cognitive skills (8.4%) - Adaptive/self-help skills (6.7%) - School readiness skills (3.4%) - Motor skills (2.2%) - Vocational skills (1.7%) - Play (0.6%) - Mental health (0.0%)​ |
| Clark & Adams 2020 | Parents identified their top priorities for autism research across three settings (home, school, community) through a survey (n=134) and Q-sort discussions (n=9):  Highest priorities home setting:   - Child health and well-being (survey) - Understanding parent, sibling, child, and family impact and stress (Q-sort)   Highest priorities school setting:   - Socialisation (Socialisation referred to peer relationships, inclusive social experiences, and support for friendship development at school) and social support (survey) - Teacher/staff education and support (Q-sort)   Highest priorities community setting:   - Community awareness and understanding of autism (survey) - Recognizing and supporting anxiety (highest priority from Q-sort)​ |
| Derguy et al., 2015 | The key outcomes parents value for their autistic children. These percentages reflect the proportion of parents who identified each area as a valued outcome during interviews:   - Material needs (60.3%) - Information needs (52.6%) - Parental guidance (43.6%) - Daily management (37.2%) - Relational support (33.3%) - Emotional support (32.1%) |
| De Korte et al., 2022 | Parents qualitatively described the following as important developmental outcomes for their autistic children:   - Improved social-communication skills - Improved well-being - Improved family cohesion |
| DuBay et al., 2018 | Latino caregivers’ perspectives on key priorities (based on qualitative data from focus groups):   - Communication development, especially spoken language - Self-regulation and behaviour management - Caregiver involvement in therapy and a strong desire for training to support their child at home - Language access and open communication with providers |
| Gormley et al., 2024 | Prioritisation by group:  Autistic non-educators (n = 34):   - Physical/mental health 69% - Self-determination 63% - Social inclusion 46% - Emotional awareness 43% - Communication 43%   Autistic educators (n = 10):   - Social inclusion 80% - Self-determination and autonomy 70% - Transitions 50% - Communication, functional/self-help skills, reducing meltdowns 40% each   Non-autistic educators (n = 65):   - Communication 75% - Functional/self-help skills 62% - Social inclusion 59% - Self-determination 45% - Social skills 45% |
| Laubscher et al., 2024 | Parents qualitatively described a range of priorities and valued outcomes for their autistic children related to communication and AAC:   - Establishing functional and reliable communication to reduce frustration, aggression, and self-harm - Enabling children to express wants, needs, emotions, and pain - Supporting independence and participation in daily activities and community life - Promoting connection and relationship-building with others - Enhancing emotional well-being for both children and families |
| Lindsay et al., 2016 | Parents' perspectives and priorities regarding their autistic children's development and support based on qualitative interviews:   - Educational progress. - Social and emotional development, with specific emphasis on peer relationships |
| Petrina et al., 2015 | Parents were asked to rate and rank six outcome priorities for their autistic children. This is how they were ranked overall:   1. Social skills 2. Emotional development 3. Friendship 4. Intellectual and academic skills 5. Physical and motor development 6. Creativity |
| Pfeiffer et al., 2016 | Key valued outcomes from the perspectives of parents from qualitative interviews:   - Future independence - Community participation - Support for sibling relationships - Participation in typical environments - Long-term planning for adult life   Key valued outcomes from the perspectives of autistic adults:   - Understanding and reducing public misconceptions - Need for acceptance and inclusive social participation - Vocational and financial independence - Opportunities for appropriate support |
| Schuck et al., 2024 | Participants (n = 214 autistic adults) provided qualitative feedback on the acceptability of common intervention goals. Each intervention goal received open-ended feedback from between 120 and 189 participants. Goals were categorised qualitatively as follows:  High-priority goals (widely endorsed by participants):   - Understanding and valuing autistic ways of being, explicitly rejecting harmful normalisation approaches. - Encouraging autonomy, self-advocacy, and interdependence rather than forced independence. - Ensuring support procedures do not cause psychological or physical harm (such as anxiety from forced masking or distress from sensory exposure). - Identifying and addressing underlying causes of behaviours rather than superficial behaviour modifications.   Low-priority goals (widely criticised by participants):   - Reducing stimming (specifically motor and vocal stimming), which participants viewed as necessary self-regulatory behaviours. - Emphasis on normalization or pursuing neurotypical standards, including goals such as increasing eye contact, reducing special interests ("fixations"), and suppressing non-harmful autistic traits |
| Sulek et al., 2024 | Outcomes were rated by autistic adults, parents, and practitioners across 47 potential outcomes.  Highest priority child outcomes:   - Improving child mental wellbeing – 0–5 years: M = 3.73, 6–12 years: M = 3.84 - Reducing behaviours that harm self/others – 0–5 years: M = 3.54, 6–12 years: M = 3.68 - Improving physical wellbeing – 0–5 years: M = 3.52, 6–12 years: M = 3.59 - Increasing inclusivity and accessibility – 0–5 years: M = 3.51, 6–12 years: M = 3.58 - Improving child understanding of self – 0–5 years: M = 3.43, 6–12 years: M = 3.72   Lowest priority child outcomes:   - Reducing sensory seeking and avoidant behaviours – 0–5 years: M = 1.38, 6–12 years: M = 1.62 - Reducing focused interests – 0–5 years: M = 1.53, 6–12 years: M = 1.69 - Social skills training – 0–5 years: M = 1.77, 6–12 years: M = 1.95 - Changing play to be more neurotypical – 0–5 years: M = 1.76, 6–12 years: M = 1.84 |
| Waddington et al., 2023 | Participants qualitatively shared their perspectives on appropriate and inappropriate goals for young autistic children.  High-priority goals:   - Enhancing quality of life - Changing the environment, not the child - Supporting diverse communication - Helping adults support children - Respecting children’s boundaries and preferences - Promoting self-determination and authentic autistic identity   Low-priority goals:   - Increasing eye contact - Increasing pretend play - Reducing stimming, special interests, or autistic behaviours - Increasing ‘neurotypical’ social skills |
| Waddington et al., 2024 | Perceptions of the appropriateness and priority of support goals for young autistic children.  Highest priority goals (ranked 1: highest–3: lowest):   - Adult supports and upskilling adult: *M = 1.11* - Reducing harmful behaviour: *M = 1.16* - Quality of life: *M = 1.20*   Lowest priority goals:   - Reducing autism characteristics: *M = 2.69* - Play skills: *M = 2.65* - Academic skills: *M = 2.64* |
